# *Cis*-trimethoxystilbene, exhibits higher genotoxic and antiproliferative effects than its isomer *trans*-trimethoxystilbene in MCF-7 and MCF-10A cell lines

**DOI:** 10.1590/1678-4685-GMB-2020-0477

**Published:** 2021-09-22

**Authors:** Natália dos Santos Gonçalves, Tamires Maria Silva Pereira de Mello, Cássia Suemi Mizuno, Saqlain Haider, Raquel Alves dos Santos

**Affiliations:** 1 Universidade de Franca, Laboratório de Genética e Biologia Molecular, Franca, SP, Brazil.; 2 University of New England, Westbrook College of Health Professions, School of Pharmacy, Department of Pharmaceutical and Social Administrative Sciences, Portland, ME, USA.; 3 University of Mississippi, School of Pharmacy, National Center for Natural Products Research, MS, USA.

**Keywords:** Stilbenes, genotoxic, cell cycle arrest, apoptosis

## Abstract

Stilbenes are a class of natural compounds with a wide variety of biological effects, such as antitumor activity. The best-known stilbene is resveratrol, whose clinical application is limited due to its low bioavailability. Methoxylated derivatives of this stilbene, including *cis*-trimethoxystilbene (*cis*-TMS) and *trans*-trimethoxystilbene (*trans*-TMS) have demonstrated more pronounced cytotoxic and anti-proliferative effects than resveratrol. Thus, the objective of this study is to evaluate and compare the cytotoxicity and antiproliferative effects of *cis*- and *trans*-TMS in MCF-7 and its normal counterpart MCF-10A. Both compounds were cytotoxic, genotoxic, and induced G2-M accumulation and cell death in the two cell lines. These results suggested that the genotoxicity of *cis*- and *trans*-TMS is involved in the reduction of cellular proliferation of MCF-7 and MCF-10A cells, but notably, such antiproliferative effects are more pronounced for *cis*- than *trans*-TMS.

## Introduction

Natural products are an important source of bioactive agents of diverse structures and biological activities. They represent the most abundant reserve of molecules for the development of drugs in clinical use (Almaliti et al., 2013). Considering the 174 new anticancer drugs discovered between 1981 and 2014, 136 were originated from or based on natural products (Cragg and Newman, 2016). New cancer therapies are needed to overcome drug resistance in some of the existing treatments, and also to find drugs that are safer and less toxic to non-tumoral cells (Lin et al., 2017). 

The stilbenes are among the natural compounds with great pharmacological potential. The most studied stilbene is resveratrol, a polyphenolic compound found in a wide variety of plant species such as grapes, blueberries, among others (Rivière et al., 2012; Elshaer et al., 2018; Mikstacka et al., 2018). Studies have demonstrated the biological potential of resveratrol such as anti-oxidant, anti-inflammatory, cardio-protective and anti-tumor activities (Paul et al., 2010; Elshaer *et al*., 2018). It has been shown that this stilbene acts at all three stages of carcinogenesis (initiation, promotion, and progression) by modulating cell division and growth control pathways, apoptosis, inflammation, angiogenesis and metastasis (Shukla and Singh, 2011). 

**Figure 1 -:**
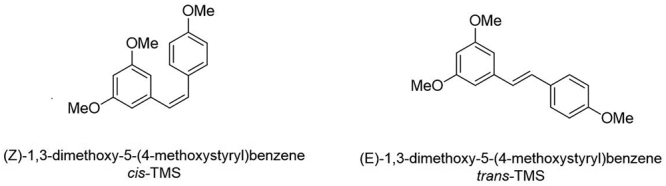
Chemical structure of *cis*- and *trans*-TMS.

Despite these promising effects, *in vivo* studies have shown that issues such as poor bioavailability due to quick metabolism can hamper the clinical use of resveratrol; this problem can be overcome using polymeric and lipid nanoparticles to improve the oral bioavailability of resveratrol (Intagliata et al., 2019). Some structural modifications of resveratrol have aimed to increase its potency (Fulda, 2010). Therefore, chemical modifications in the resveratrol structure are related not only to overcome the bioavailability but to improve its biological activity (Tian and Liu, 2020). Structure-activity relationship studies have shown that the substitution of the hydroxyl groups of resveratrol by methoxy groups improves the antitumoral potential of the compounds and increases cytotoxicity against lung and colon carcinoma cell lines (Fulda, 2010; Paul et al., 2010). Thus, two methoxylated derivatives of resveratrol have been synthesized and tested: the (*Z*)-1,3-dimethoxy-5-(4-methoxystyryl)benzene (*cis*-TMS) and (*E*)-1,3-dimethoxy-5-(4-methoxystyryl)benzene (*trans*-TMS) (Figure 1). Some resveratrol derivatives, including trimethoxystilbene acted as a tubulin depolymerizing agent inducing mitotic delay and mediating the production of multipolar spindles that caused the mitotic catastrophe and cell death in HeLa cells (Traversi et al., 2017). *Cis*-TMS also increased the micronucleus frequency in CHO-K1 and HepG2 cells, demonstrating its genotoxic effects (Mizuno et al., 2017). As well known, cancer treatment is based on the use of genotoxic agents (Connor, 2015). Despite the evidence that *trans*-TMS blocked the epithelial-mesenchymal transition of the tumoral breast cell line MCF-7, no current data is reporting the antiproliferative effects of *cis*-TMS in this cell line. In the present study, we tested and compared the antiproliferative effects of *cis*- and *trans*-TMS in the mammary adenocarcinoma cell line (MCF-7) and its standard counterpart (MCF-10A).

## Material and Methods

### Synthesis of stilbenes

The stilbenes were synthesized as previously published (Mizuno et al., 2008). Shortly, to a cold solution (-78 °C) of phosphonium salt (1.0 equivalent) in anhydrous THF was added *n*-butyllithium (1.6 mol in hexane, 1.0 equivalent) and the resulting solution was stirred under inert atmosphere for 2 h. A solution of aldehyde (1.0 equivalent) in THF was added dropwise, and the mixture was stirred for 12 h at room temperature. The resulting suspension was poured into water and extracted with dichloromethane. The organic phase was combined and dried over MgSO_4_, and concentrated under reduced pressure. The crude product was purified through automated flash purification elution with hexane/ethyl acetate.

*Trans*-TMS. ^1^H NMR(CDCl_3_, 300 MHz): δ 3.83 (s, 9H); 6.37 (t, 1H, *J* = 2.1 Hz); 6.65 (d, 2H, *J* = 1.8 Hz); 6.87-6.89 (m, 2H); 6.92 (d,1H, *J* = 5.1 Hz); 7.04 (d, 1H, *J* = 16.2 Hz); 7.45 (d, 2H, *J* = 6.9 Hz). ^13^C NMR (CDCl3, 75 MHz): δ 55.2 (3C), 99.7, 104.2 (2C), 114.2 (2C), 126.5, 127.8 (2C), 129, 130, 139.6, 159.4, 161.0 (2C). LC-MS m/z 270.19. (M+H)^+^.

*Cis*-TMS. 1H NMR(CDCl_3_, 500 MHz): δ 3.71 (s, 6H); 3.81 (s, 3H); 6.36 (t, 1H, *J* = 5 Hz); 6.47-6.50 (m, 3H); 6.57 (d, 1H, *J*= 10 Hz); 6.81 (d, 2H, *J*= 10 Hz); 7.26 (d, 2H, *J*= 10 Hz). ^13^C NMR(CDCl_3_, 125 MHz): δ 55.2 (3C), 99.6, 106.6 (2C), 113.5 (2C), 128.7, 129.5, 130.2, 130.3 (2C), 139.5, 158.7, 160.5 (2C). LC-MS m/z 271.136. (M+H)^+^.

### Cell culture and treatment solutions

MCF-7 and MCF-10A cells, obtained from Cell Bank of Rio de Janeiro, were cultured in complete medium containing DMEM+HAMF10 medium (1:1, v/v), supplemented with 10% of fetal bovine serum and antibiotic mixture penicillin/streptomycin (10 mL/L) plus kanamycin sulfate (10 mg/L) using culture bottles of 25 mm^2^ or multiple well plates when necessary. The cells were incubated at 37 °C under an atmosphere saturated with 5% of CO_2_. 

The *cis*-TMS and *trans*-TMS were synthesized as previously reported (Mizuno et al., 2008). The compounds were dissolved in dimethyl sulfoxide (DMSO) into a stock concentration of 0.01 M. The final concentration of DMSO in culture was 0.1%. Doxorubicin (DOX, 0.5 µM) (Sigma-Aldrich) was a positive control. All reagents were dissolved just before use and protected from light.

### Cytotoxicity assay - XTT

Cytotoxicity assay used the Cell Proliferation Kit (Roche Applied Science). On a 96 wells plate, 10^4^ cells/well were seeded and incubated in an atmosphere of 5% CO_2_ at 37 °C for 24 h. After this period, the cells were exposed to different concentrations of *cis*-TMS and *trans*-TMS (7.8 to 1000 µM), 0.5 µM of doxorubicin, or 0.1% of DMSO. Doxorubicin and DMSO were positive and vehicle control, respectively. The cells were incubated for an additional 24 h and, the plate was washed with phosphate buffer saline (PBS 1x). The plate was then incubated with DMEM without phenol red plus the XTT/electron solution for 4 h. Total absorbance was measured at 492 and 690 nm. The number of viable cells was directly proportional to the absorbance and the percentage was compared with the negative control.

### Clonogenic survival assay

The clonogenic assay was according to Franken et al. (2006). Briefly, 200 cells/well were seeded in six wells plates containing 2 mL of complete medium. After four hours, the cells were treated with different concentrations of *trans*-TMS (2.5 µM, 5 µM, and 10 µM) and *cis*-TMS (0.3125 µM, 0.625 µM, 1.25 µM, 2.5 µM, 5 µM, and 10 µM) for 24 h. After this period, each well was washed with PBS 1x, completed with medium, and allowed to grow for 7-14 days, when the colonies were visible. The cells were fixed with a mixture of methanol/acetic acid/water (1:1:8, v/v/v) for 30 min and stained with Giemsa/Sörensen phosphate buffer (1:20, v/v) for 15 to 30 min. The colonies were counted using a stereomicroscope, and the cell survival fraction was calculated as a percentage compared to the negative control.

### Genotoxicity - comet assay

The alkaline comet assay evaluated the genotoxicity. Ten thousand cells/well were seeded in 12 wells plates, and after 24 h, treated with different concentrations of *trans*-TMS and *cis*-TMS for four hours. After treatments, 20 µL of cell suspension was mixed with 200 µL of low melting point agarose (0.5%). This cell suspension/agarose mixture was poured on the previously coated slides with agarose (1.5%) and covered with a coverslip. After solidification, the coverslip was removed, and the slides were placed in a cold lysis solution (NaCl 2.5 M, Na_2_EDTA 100 mM, Tris 10 mM, Triton X-100 1%, and DMSO 10%, pH 10.0) for at least two hours. Then, they were incubated in alkaline buffer (NaOH 0.3 M, and Na_2_EDTA 1 mM, pH >13) at 4 °C for 20 min and submitted to electrophoresis for 30 min at 25 V 300 mA. The slides were incubated in a neutralization buffer for 15 min (Tris-HCl 0.4 M, pH 7.5) and fixed with 15% CCl_3_COOH, 5% ZnSO_4_ 7 H_2_O and 5% C_3_H_8_O_3_ for 10 min. All slides were stained using silver nitrate as described by Nadin et al. (2001). A total of 150 nucleoids were analyzed per treatment according to the level of DNA migration (Tice et al., 2000).

### Analysis of cell cycle

For cell cycle kinetics, 5x10^4^ cells/wells were seeded in 12 wells plate, and after 24 h, the cells were treated for an additional 24 h with 2.5 µM of *cis*-TMS or 10 µM of *trans*-TMS. The cells were harvested and stained with propidium iodide to determine the different phases of the cell cycle by flow cytometry using the Flow Cytometry BD FACSCanto (BD Biosciences, USA).

### Determination of apoptotic cells fraction

The fraction of apoptotic cells was determined by flow cytometry. For this assay, 2x10^4^ cells/well were seeded in 12 wells plate. After 24 h, the cells were treated with 2.5 µM of *cis*-TMS or 10 µM of *trans*-TMS and incubated for 24 h. Then, the cells were harvested and processed according to the manufacturer’s instructions using the Dead Cells Apoptosis Kit (Thermo Fisher Scientific, USA). The apoptotic cell frequency was determined using the Guava® EasyCyte™ (GE Healthcare, NJ, USA). By this procedure, early apoptotic cells are Annexin V-positive and PI-negative (Annexin V-FITC+/PI−), whereas late (end-stage) apoptotic cells are Annexin V/PI-double-positive (Annexin V-FITC+/PI+) (Wlodkowic et al., 2011).

### Statistical analysis

All statistical analyses were performed using GraphPad Prism 5.0 software. Analysis of variance (ANOVA), followed by Bonferroni post-test, compared the experimental groups. Statistically significant values were those with p ≤ 0.05, and the results were reported as means and standard deviations (SD) of three independent experimental samples. Negative control (NC) and vehicle control (VC) did not exhibit statistical differences; therefore, treatments were compared to the vehicle (VC).

## Results

### The cytotoxicity of *cis* and *trans*-TMS

**Figure 2 -:**
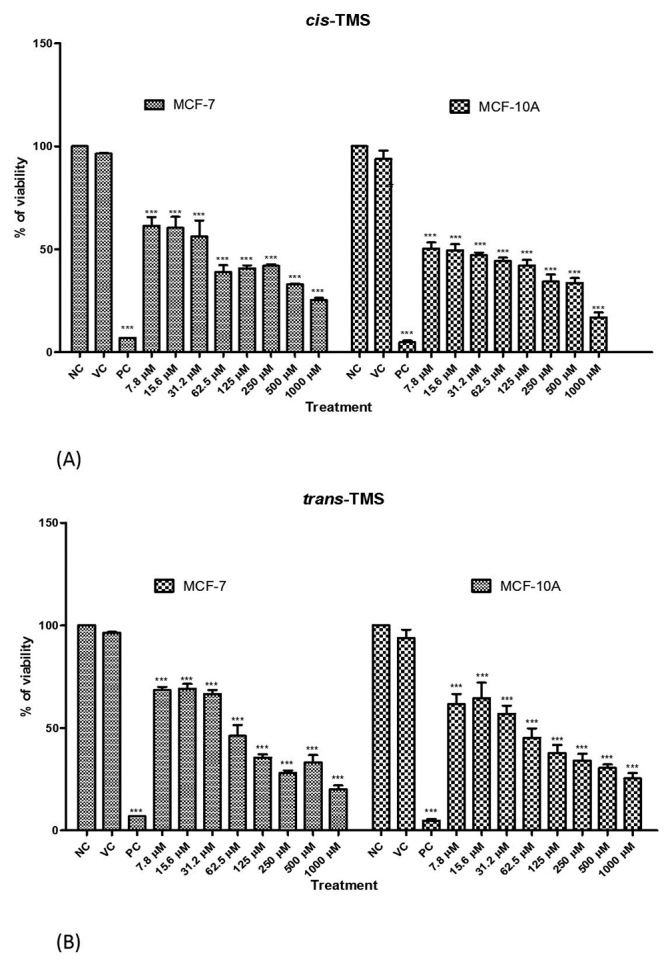
Percentage of cell viability obtained by XTT assay in MCF-7 and MCF-10A cells treated with different concentrations of *cis*- (A) or *trans*-TMS (B). NC: negative control; VC: vehicle control; PC: positive control; *** *P* < 0.001 (*vs* NC).

Figures 2A and B exhibit the results of cell viability analysis obtained by XTT assay after 24 h of treatment with different *cis-* and *trans-*TMS concentrations. Both compounds caused a significant reduction of cell viability at all tested concentrations in both cell lines (*p* < 0.0001 *vs*. VC). Both substances showed concentration-dependent cytotoxic activity and were not selective between MCF-7 and MCF-10A cells since no statistical differences were detected between both cell lines. The calculated inhibitory concentration (IC50) was 42.2 and 59.5 µM for *cis*- and *trans-*TMS in MCF-7 and, 16.2 µM and 45.7 µM in MCF-10A for *cis-* and *trans*-TMS, respectively*.*

**Figure 3 -:**
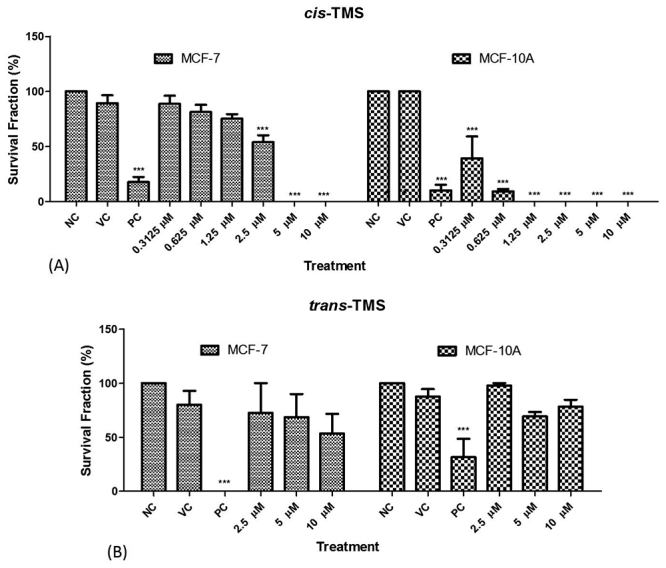
Survival fraction obtained in MCF-7 and MCF-10A cells treated with different concentrations of *cis*- (A) or *trans*-TMS (B). NC: negative control; VC: vehicle control; PC: positive control; *** *P* < 0.001 (*vs* NC).

To analyze the long-term cytotoxicity of the two stilbenes, we performed the clonogenic survival assay. The chosen concentration range was based on the XTT results described above and on the concentrations used to determine the survival fraction as we previously described (Mizuno et al., 2017). Therefore, the concentrations of 0.31 µM, 0.62 µM, 1.25 µM, 2.5 µM, 5 µM, and 10 µM were chosen for the assay with the *cis*-TMS, while for the *trans*-TMS, the concentrations of 2.5 µM, 5 µM, and 10 µM were selected. Treatment of the cells with *cis*-TMS significantly reduced the survival fraction of MCF-7 cells in concentrations higher than 2.5 µM (*p* < 0.001) (Figure 3A). In MCF-10A, the *cis*-TMS reduced the survival fraction in all tested concentrations (*p* < 0.001) (Figure 3A). Treatments with *trans*-TMS showed no significant reduction in cell survival fraction at all the tested concentrations in both cell lines (Figure 3B).

### DNA damage analysis

**Figure 4 -:**
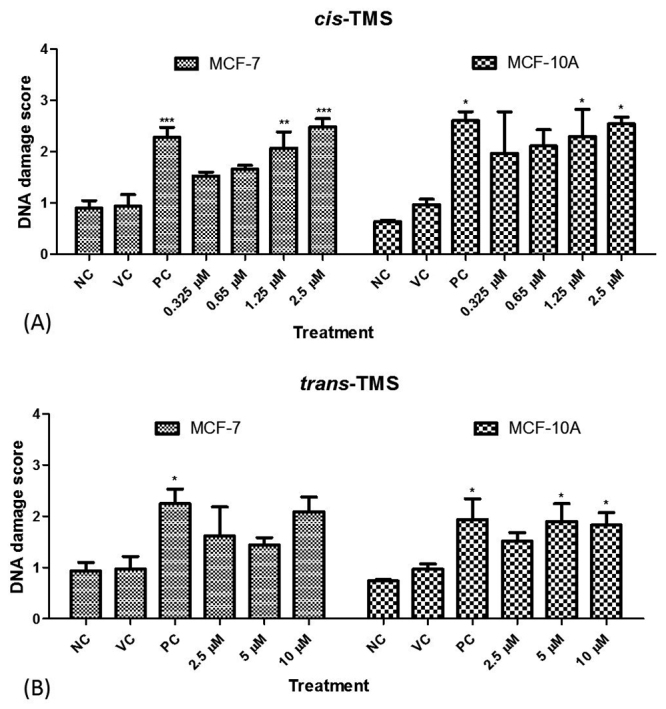
DNA damage obtained by comet assay in in MCF-7 and MCF-10A cells treated with different concentrations of *cis*- (A) or *trans*-TMS (B). NC: negative control; VC: vehicle control; PC: positive control; * *P* < 0.05 (*vs* NC); ** *P* < 0.01 (*vs* NC); *** *P* < 0.001 (*vs* NC).

The genotoxicity was analyzed using the alkaline version of comet assay 4 hours after treatment with *cis*- or *trans*-TMS in both cell lines (Figure 4). Treatments with 1.25 and 2.5 µM *cis*-TMS significantly increased the DNA damage score in MCF-7 (*p* < 0.01 and 0.001, respectively) and MCF-10A (*p* < 0.05) (Figure 4A), while *trans*-TMS at 5 and 10 µM increased the DNA damage score only in MCF-10A cells (*p* < 0.05) (Figure 4B).

### Cell cycle and cell death analysis

**Figure 5 -:**
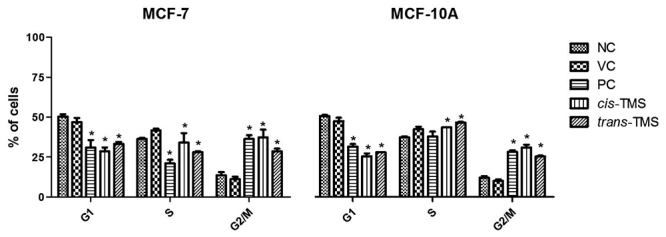
Distribution of MCF-7 (A) and MCF-10A (B) in different phases of cell cycle after treatment with *cis*-TMS (2.5 µM) or *trans*-TMS (10 µM). NC: negative control; VC: vehicle control; PC: positive control; * *P* < 0.05 (*vs* NC).

Figure 5 exhibits the distribution of cells in different phases of the cell cycle after treatment with 2.5 µM of *cis*- or 10 µM of *trans*-TMS. The treatment with *cis*- or *trans*-TMS significantly reduced the frequency of G1 cells and notably increased the frequency of G2-M cells in both cell lines compared to the negative control (*p* < 0.05). No statistical differences in response to the treatments were observed comparing the MCF-7 and MCF-10A cells.

**Figure 6 -:**
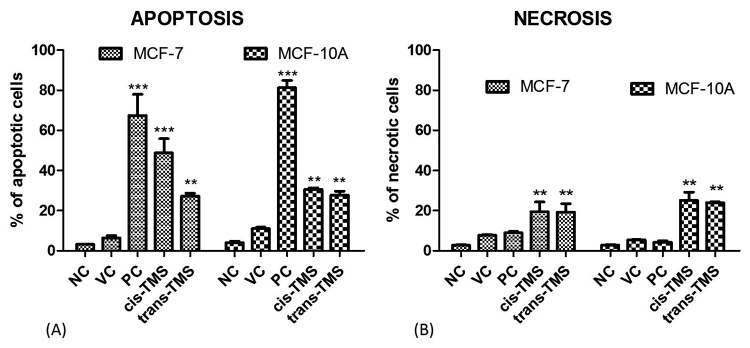
Frequency of apoptotic (A) and necrotic (B) cells 24 h after treatment with *cis*-TMS (2.5 µM) or *trans*-TMS (10 µM). NC: negative control; VC: vehicle control; PC: positive control; ** *P* < 0.01 (*vs* NC); *** *P* < 0.001 (*vs* NC).

Figure 6 shows the results of cell death rates after treatment with *cis*- or *trans*-TMS. The frequency of apoptosis (Figure 6A) and necrosis (Figure 6B) was significantly higher in MCF-7 and MCF-10A cells treated with *cis*- or *trans*-TMS than in the negative control. The frequency of apoptosis in MCF-7 was 48.7 and 27.1% after treatment with *cis*- or *trans*-TMS, respectively, while MCF-10A exhibited 30.5 and 27.6% of apoptosis, respectively (Figure 6A). No difference in the frequency of necrotic cells between both cell lines was observed (Figure 6B). 

## Discussion

In the present study, the antiproliferative effects of two stilbene derivatives, *cis*- and *trans*-TMS, were evaluated and compared in the tumoral breast cell line MCF-7 and its normal counterpart MCF-10A. Previous cytotoxicity screening of these stilbenes in different cancer cell lines revealed MCF-7 as the most sensitive to the treatment with *cis*- and *trans*-TMS (non-published data).

The treatment with *cis*- and *trans*-TMS reduced the cellular viability of both cell lines, as demonstrated by the XTT assay. The calculated IC_50_ revealed higher cytotoxicity of *cis*- than *trans*-TMS. However, while relative IC_50_ values may have predictive value in comparing a series of compounds with a similar mode of action, absolute IC_50_ values do not predict cell survival (Brown and Wouters, 1999). This is clearly demonstrated by the survival fraction obtained after treatment with *cis*- or *trans*-TMS in both cell lines in the present study. Although the value of short-term cytotoxicity assays is based on tetrazolium salts to reveal the fraction of still metabolically active cells, these non-clonogenic assays may underestimate the cytotoxicity of a compound. In addition, these assays can overestimate the cytotoxicity by not consider cells with reversible damage or resistance to the cytotoxic agent tested (Adan et al., 2016). Loss of tumor cell reproductive viability, measurable in many cell lines by clonogenic survival assays, is the most direct method of measuring a drug’s cytotoxic activity (Puck and Marcus, 1955). Therefore, the clonogenic survival assay measures the long-term cytotoxic effects of a molecule by measuring the proliferative capacity of a single cell to form a clone and thereby produce a viable colony (Sumatran, 2011).

According to our results, even low concentrations of the *cis*-TMS inhibited the colony formation, while *trans-*TMS did not reduce the survival fraction to statistically significant levels. The reduction of the survival fraction after *cis*-TMS treatment could be related to its genotoxicity. In the presence of DNA damage that cannot be repaired, p53 protein may activate the DNA damage response pathway modulating the cell cycle progression or triggering apoptosis to prevent the replication of damaged chromosomes (Williams and Schumacher, 2016). Both *cis*- and *trans*-TMS increased the extension of DNA damage in both cell lines, but while the *cis*- was genotoxic to both cell lines, the *trans*-TMS increased the DNA damage score only in MCF-10A and in concentrations two or four times higher than the *cis*. 

Since both *cis*-TMS and *trans*-TMS displayed cytotoxic and genotoxic effects, the ability of these two substances to modulate the cell cycle kinetics and induce cell death was investigated. Both stilbenes caused an accumulation of cells in the G2 phase and increased the frequency of apoptosis in MCF-7 and MCF-10A. The accumulation of both cell lines in the G2-M phase may be related to the genotoxicity of the substances since the induction of DNA damage during the S phase can lead to the G2-M cell cycle arrest and subsequent death by apoptosis. Treatment of hepatoma-derived cell lines with 1 µM of *cis*-TMS during 48 h induced accumulation in the G2-M phase with a concomitant reduction in G1 and S phases (Nguyen et al., 2016). These effects were attributed to the downregulation of total cyclin-dependent kinase 1 (CDK1) with concomitant induction of p21^Cip/Waf1^ expression. *Trans*-TMS at 80 µM also stalled HCT116 cancer cells in G2-M with subsequent cell death (Traversi et al., 2019). There is strong evidence in the literature that the effects of TMS inhibit tubulin polymerization during the S phase, which leads to the destabilization of microtubule activity and consequent cell cycle delay and death (Chabert et al., 2006; Traversi *et al*., 2016; Traversi *et al.*, 2017). 

Our results demonstrated that *cis*- and *trans*-TMS induced apoptosis in both cell lines. Apoptosis can occur in response to different situations, including after cell damage by genotoxicity caused by chemotherapeutic agents (Grivicich et al., 2007). Our data suggest that the genotoxic effects of *cis*- and *trans*-TMS are related to cell cycle arrest and apoptosis. 

When considering the overall results of the present study it is clear that *cis*- and *trans*-TMS are not selective between MCF-7 and MCF-10A and that the cellular response to *cis*-TMS occurs in concentrations four times lower than *trans*-TMS. *Cis*- and *trans*-TMS are geometric isomers. They are non-superimposable, non-mirror images of each other. They result from the restricted rotation of a carbon-carbon bond. The two enantiomers of a chiral drug may differ significantly in their bioavailability, rate of metabolism, metabolites, excretion, potency, selectivity for receptors, transporters, and enzymes, and toxicity, leading to the choice for a single-enantiomer drug with more straightforward and more selective pharmacologic profiles, improved therapeutic indices and simpler pharmacokinetics (McConathy and Owens, 2003). 

In conclusion, the present results suggest that the genotoxicity of both compounds may be involved in the antiproliferative effects observed in MCF-7 and MCF-10A cell lines. However, this effect is considerably more pronounced for *cis*-TMS.
